# Concrete Durability after Load Damage and Salt Freeze–Thaw Cycles

**DOI:** 10.3390/ma15134380

**Published:** 2022-06-21

**Authors:** Jiguo Zhou, Guihua Wang, Peng Liu, Xuefeng Guo, Jun Xu

**Affiliations:** 1 College of Civil Engineering, Baicheng Normal University, Baicheng 137099, China; wangguihua@bcnu.edu.cn (G.W.); bcsyliupeng@bcnu.edu.cn (P.L.); guoxuefeng@bcnu.edu.cn (X.G.); 2 College of Civil Engineering and Architecture, Jiangsu University of Science and Technology, Zhenjiang 212003, China; xujun@just.edu.cn

**Keywords:** compressive strength, concrete durability, erosion property, pore characteristics, quick freeze–thaw cycle

## Abstract

To determine how the performance of concrete changes after initial load damage and salt freezing, concrete samples were first subjected to loading and unloading, and were then put into salt solutions to carry out rapid freeze–thaw cycle (FTC) experiments. Salt solutions were created based on the saline soil of western Jilin, China, for use in salt freeze–thaw testing. This determined the change law of the compressive strength and the dynamic elastic modulus (DEM). Additionally, low-field nuclear magnetic resonance technology and a scanning electron microscope were applied to investigate the pore characteristics and microstructure of concrete samples after FTCs. This study found that when the concrete specimens were subjected to an initial load of 0.3*f* under 50 FTCs, the loss in the compressive strength increased by 24% when the concrete was subjected to freeze–thaw cycles in freshwater and increased by 24% when concrete was subjected to freeze–thaw cycles in a 6.8% composite salt solution compared with the specimens without the initial load. When the concrete was subjected to FTCs in a 6.8% composite salt solution 50 times, the loss in the compressive strength increased by 110% for concrete without an initial load and increased by 109% when the concrete was subjected to an initial load of 0.3*f* compared with the specimens under FTCs in freshwater. The persistent effect of the FTCs also aggravated chloride ion erosion in the concrete, which gradually reduced the concrete’s permeability resistance. Internal pores in the concrete, especially the proportion of above-medium-sized pores, gradually increased along with the increase in the number of FTCs. There is a good linear correlation between the change rule of compressive mechanical properties and the change rules of mass, DEM, and pore characteristics inside the concrete under rapid FTCs in different salt solutions.

## 1. Introduction

Concrete-engineered buildings are frequently subjected to multiple coupling effects. Therefore, concrete durability under those effects has always been a research focus [1,2,3]. The durability of concrete will decrease when concrete is under the combined action of salt corrosion and the freeze–thaw cycle, which will reduce the service life of concrete structures [4,5,6,7]. The effect of environmental freeze–thaw cycles (FTCs) will increase the permeability of concrete, including its water absorption and ion permeability [8]. The strength and stiffness of concrete will decrease and seriously affect the bearing capacity of concrete structures when they are subjected to long-term corrosion [8]. Comparative studies have shown that damage to concrete subjected to FTCs in a salt solution is more severe than in freshwater [9,10]. Research on the durability of concrete subjected to salt freezing has been a focus in recent years [11].

The hydrostatic, osmotic, and expansion pressures generated in concrete during FTCs in freshwater form cracks and reduce concrete strength. In a salt solution, salt crystal pressures are very destructive to concrete subjected to FTCs. Additionally, multiple FTCs in a salt solution will lead to more crystallization, osmotic pressures, and cracking damage in concrete [12]. The action of FTCs not only leads to concrete mass and strength losses, but also affects the transmission of chloride ions in concrete structures [13,14]. Solving concrete’s practical structural durability problems under FTCs requires studying both material properties and environmental characteristics. Analyzing the mechanism of concrete FTCs can be carried out from the perspectives of the compressive strength, microscopic pore structure, and composite changes. Such an analysis has established the durability characteristics and a damage model for concrete subjected to FTCs [15,16,17]. The research shows that the diffusion law of chloride ion in concrete satisfies Fick’s second law when concrete is under the coupling action of chloride erosion and freeze–thaw conditions [18]. The sulphate attack will cause the cracking of concrete and accelerate the external corrosive medium, such as water and gas infiltration into concrete structure, which will lead to the destruction of the microstructure and the reduction in the macro-performance of the concrete [19]. Samaha and Hover [20] reported that microcracking in concrete at stress levels below 75% of the compressive strength did not affect the mass transport properties of concrete. The chloride permeability of concrete (after it was unloaded) appears to be influenced by the occurrence of a certain stress level known as the critical stress [21].

Researchers have conducted a lot of effective studies on the penetration and diffusion behavior of chloride ions in concrete materials, which have mostly concentrated on the effect of load, salt solution soaking, and wet–dry cycles in salt solution as well as studies on the ion diffusion in saturated or unsaturated concrete structures. Experimental studies promote the understanding of chloride diffusion in concrete under loads [22]. The concrete structures in practical engineering usually work under the combined action of loads, environmental influence, and many other factors. Multi-factors cause the breaking mechanism to be more complicated. Scholars worldwide have carried out systematic studies on the durability damage mechanism, degradation law, and durability repair and promotion, and have obtained many meaningful results which include single- and multi-factor coupling actions and research on materials and structures [23].

The changes in the chloride penetration behavior and mechanical properties of concrete are influenced by many factors. In the previous studies, fewer attempts have been made to study the coupling effect of initial loads and FTCs on the changes in the chloride ion penetration and mechanical properties of cement concrete. Further research is needed to study the relationship between chloride diffusion, mechanical properties, and the microstructure of concrete structures. The research combining macro mechanical properties with pore changes inside the concrete structure to reveal the mechanism of load influence is very meaningful [24]. In this study, experiments were carried out to investigate the effects of the initial load damage of concrete samples that were then subjected to FTCs in salt solutions. The mechanical properties, physical properties, microscopic pore structure, and component changes in the concrete under FTCs were analyzed to find the variation law of the durability and reveal the relationship between the macroscopic properties and microscopic pores of concrete subjected to FTCs in salt solutions with initial load damage.

## 2. Raw Materials and Experimental Scheme

### 2.1. Raw Materials

This study’s concrete cementitious materials were ordinary Portland cement PO 42.5, fine concrete aggregate with a fineness modulus of 2.2, river sand, and a coarse concrete aggregate of graded gravel with a diameter of 4.75 to 26.5 mm. The concrete mixing proportion and compressive strengths of 7 and 28 d are shown in Table 1.

### 2.2. Test Methods

The experiments were focused mainly on the effect of FTCs on the durability of concrete after initial load damage. The 100 mm × 100 mm × 100 mm cubic samples were fabricated for testing compressive strength, and the 100 mm × 100 mm × 400 mm rectangular samples were fabricated for testing the dynamic elastic modulus (DEM). The tested concrete blocks were fabricated following Chinese Standard GB/T 50081-2019, the Standard for Test Methods of Concrete Physical and Mechanical Properties [25]. The cement concrete specimens were maintained in a standard curing room with a humidity of no less than 95% and a temperature of 20 ± 2 °C after pouring operations. The experiments began by carrying out the application of the initial load and fast FTCs on the cement concrete specimens when the curing age was 28 days. The initial load applied was quantitatively characterized by stress level *R*_s_, where *R*_s_ = σ/*f*, with σ denoting the stress applied on the concrete specimens during the test and *f* denoting the compressive strength of the concrete specimen at 28 days under standard maintenance. After being maintained under standard conditions for 28 d, the concrete samples were subjected to initial loading and unloading, with respective *R*_s_ of 0, 15%, and 30%. Then, the samples were subjected to FTC action. We tested the sample loads and unloaded the stress level loads five times to realistically simulate the effect of those loads on concrete materials [26].

After the initial applied loading and unloading, the samples were immersed in either freshwater, a 3.4% salt solution, or a 6.8% salt solution for rapid FTCs in accordance with Chinese Standard GB/T 50082–2009, the Standard for Test Methods of the Long-Term Performance and Durability of Ordinary Concrete [27]. The mechanical properties test of the concrete specimens was carried out according to Chinese Standard GB/T 50081–2019 [25], and the physical properties test of the concrete specimens was carried out according to Chinese Standard GB/T 50082–2009 [27]. To ensure its applicability to real-life engineering, the experiment simulated the compositional characteristics of saline soil in western Jilin province, China [12]. That area’s salt-frozen environment was emulated by preparing a salt solution for the FTCs using sodium chloride (NaCl), sodium sulphate (Na_2_SO_4_), and sodium bicarbonate (NaHCO_3_) in proportion. The overall experimental scheme is shown in Table 2. Tap water was applied for salt solution matching, and the salt concentration in the freshwater was negligible. A WAW-600 press machine, a DT-20 ammunition instrument, an HC-RCTF quick tester, and an MRE-60 low-field NMR were used to test the compressive strength, the dynamic elastic modulus, the chloride concentration, and the pore characteristics of the concrete material, respectively.

## 3. Mechanical Performance of Concrete Samples

### 3.1. Compressive Strength Change

**Figure 1:**
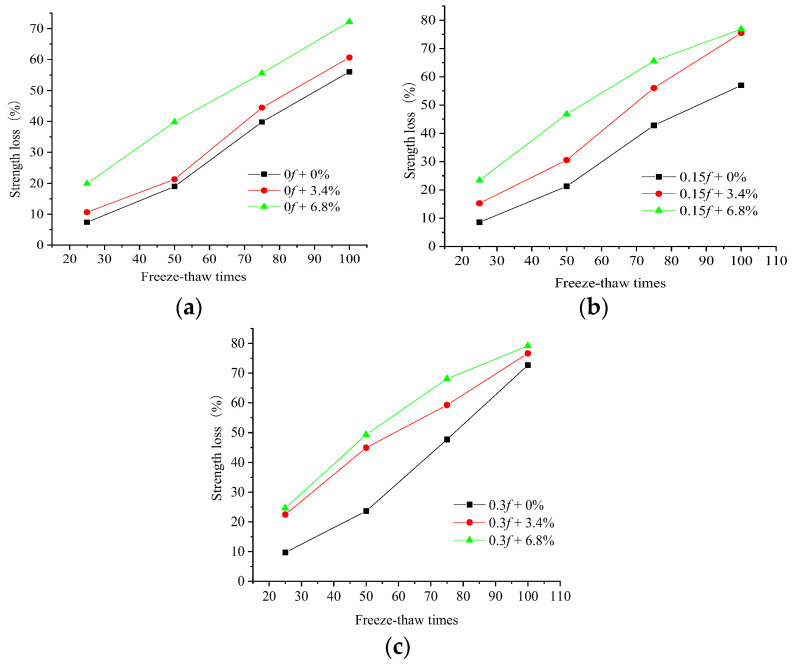
Compressive strength of concrete samples. (**a**) Without the initial load. (**b**) Initial load of 0.15*f*. (**c**) Initial load of 0.3*f*.

The concrete samples were subjected to 0, 15%, and 30% stress levels, then placed in water, 3.4%, or 6.8% concentrations of salt solution for the rapid freeze–thaw experiment. The compressive strength, mass, and DEM were tested after every 25 FTCs. A WAW-600 press (Guizhou Sunpoc Tech Industry Co., Ltd., Guiyang, China) was applied for the compressive strength test. The change law of the compressive strengths of the concrete with various numbers of FTCs is shown in Figure 1.

The variation in the compressive strength of the concrete samples was compared with the number of FTCs, and it was found that the compressive strength decreased almost linearly with the increase in the number of salt FTCs. The compressive strength decreased as the number of FTCs, the amount of the initial loads, and the salt solution concentration increased. The initial load affected the concrete mechanism properties and aggravated the damage of the freeze–thaw action on the concrete. The damage of the freeze–thaw action in a salt solution exceeded that in freshwater, and the salt concentration also greatly influenced the freeze–thaw damage.

The initial stress action of 0.3*f* caused a larger loss in the compressive strength of the concrete specimens than no initial load. Moreover, the loss in the compressive strength increased by 24% after 50 FTCs and by 30% after 100 FTCs when concrete was under FTCs in freshwater, and the loss in the compressive strength increased by 24% after 50 FTCs and by 10% after 100 FTCs when concrete was under FTCs in the 6.8% composite salt solution. The FTCs in the 6.8% composite salt solution caused a larger loss in the compressive strength of the concrete specimens than FTCs in freshwater. The loss in the compressive strength increased by 110% after 50 FTCs and by 29% after 100 FTCs when the concrete did not have an initial load, and the loss in the compressive strength increased by 109% after 50 FTCs and by 10% after 100 FTCs when concrete under FTCs in the 6.8% composite salt solution was subjected to an initial stress action of 0.3*f*. The coupling action of an initial stress of 0.3*f* and FTCs in the 6.8% composite salt solution aggravated the damage of FTCs to concrete. When concrete under FTCs in the 6.8% composite solution was subjected to an initial load of 0.3*f*, the loss in the compressive strength of the concrete was 159% larger after 50 FTCs and 41% larger after 100 FTCs than concrete under FTCs in freshwater without an initial load.

**Figure 2:**
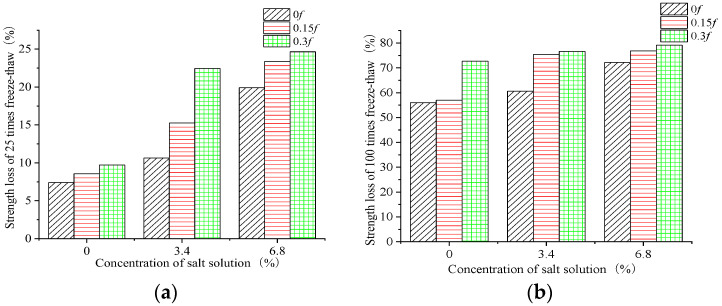
Change in the compressive strength with freshwater and two concentrations of salt solution. (**a**) After 25 freeze–thaw cycles. (**b**) After 100 freeze–thaw cycles.

**Figure 3:**
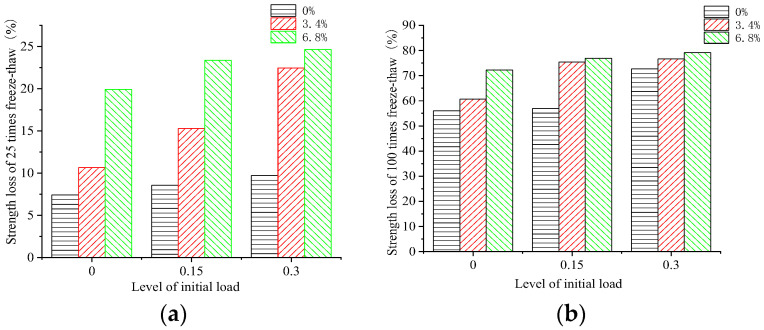
Change in the compressive strength with three levels of initial load. (**a**) After 25 freeze–thaw cycles. (**b**) After 100 freeze–thaw cycles.

Figure 2 and Figure 3 compare 25 and 100 FTCs and show the change rule of the compressive strength of the concrete samples subjected to FTCs in freshwater and two salt solutions after two initial loads.

In the FTCs, the initial loads led to an increase in the number and diameter of pores inside the concrete samples. The initial loads led to a higher freezing pressure inside the concrete, and the initial load damage to the concrete reduced the concrete’s compressive strength under the freeze–thaw action. The concrete was also subjected to salt crystallization pressure during the salt solution FTCs. At the same time, the salt solution increased the internal saturation of the concrete structure, which led to more severe damage than in freshwater. Therefore, more FTCs, a greater initial load level, and a higher salt solution concentration resulted in greater internal damage and compressive strength loss. The compressive strength loss in the 6.8% composite salt solution exceeded that under FTCs in freshwater with an initial load of 0.3*f*, and the difference in compressive strength loss was greater at the beginning of the FTCs. From the compressive strength loss of the concrete samples after 25 FTCs, it can be seen that the compressive strength loss under the combined action of the initial 0.15*f* load and FTCs in the 3.4% composite salt solution was slightly greater than that under the combined action of an initial 0.3*f* load and FTCs in freshwater. It was concluded that when the concrete had a load with a low stress level before the FTCs, the salt solution concentration greatly influenced the early stage of FTCs. Additionally, the coupled actions of minor load damage and FTCs in a salt solution had more influence on the compressive strength of the concrete than a large load and FTCs in freshwater.

### 3.2. Dynamic Elastic Modulus Change

**Figure 4:**
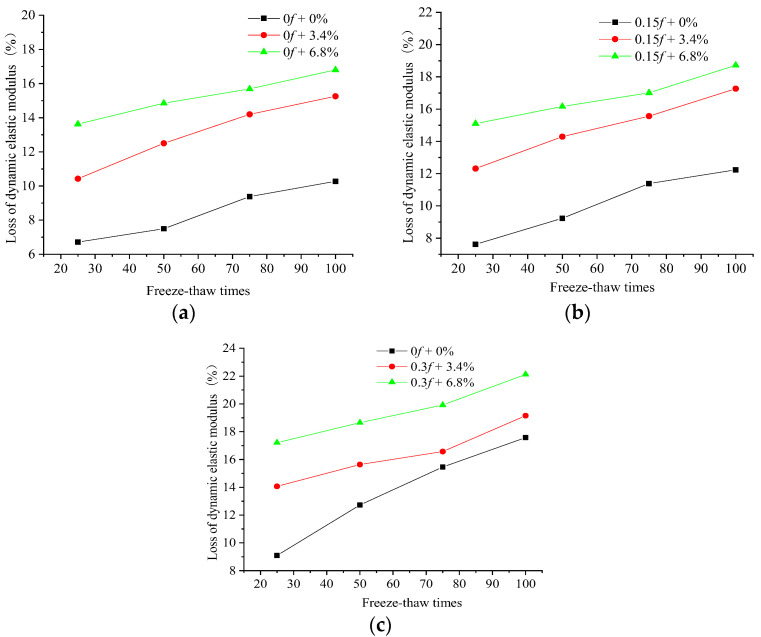
Dynamic elastic moduli of concrete samples in freshwater and two salt solutions. (**a**) Without an initial load. (**b**) With an initial load of 0.15*f*. (**c**) With an initial load of 0.3*f*.

The dynamic elastic moduli of the 100 mm × 100 mm × 400 mm rectangular concrete samples were tested by a DT-20 DEM instrument. The variation rule of the DEM of the concrete samples subjected to different initial load damage and numbers of FTCs in freshwater, and the 3.4% and 6.8% composite salt solutions is shown in Figure 4.

It can be seen that the dynamic elastic moduli decreased with an increasing number of FTCs. The DEM loss of the concrete samples subjected to different initial loads, then put under FTCs in different salt solutions, varied almost linearly with the number of FTCs. Additionally, the loss of the DEM of the concrete under FTCs was proportional to the initial stress level and the concentration of the salt solution. By comparing the change in the DEM of concrete after 25 FTCs in freshwater and two salt solutions, it is apparent that the effect of the FTCs in a salt solution on the concrete’s DEM exceeded the effect of the FTCs in freshwater. The loss of the DEM of the concrete was significant in the early FTCs in the salt solution, and the loss of the DEM of concrete subjected to an initial stress of 0.15*f* and then put under FTCs in the 3.4% composite salt solution was more significant than that subjected to an initial stress of 0.3*f* and then put under FTCs in freshwater.

The initial 0.3*f* stress action caused a larger loss in the DEM of concrete than no initial load. The loss in the DEM increased by 69% after 50 FTCs and by 71% after 100 FTCs when concrete was put under FTCs in freshwater, and the loss in the DEM increased by 25% after 50 FTCs and by 31% after 100 FTCs when the concrete was put under FTCs in a 6.8% composite salt solution. The FTCs in the 6.8% composite salt solution caused larger loss in the DEM of concrete than FTCs in freshwater. The loss in the DEM increased by 98% after 50 FTCs and by 60% after 100 FTCs when the concrete did not have an initial load, and the loss in the DEM increased by 50% after 50 FTCs and by 25% after 100 FTCs when the concrete under FTCs in the 6.8% composite salt solution was subjected to an initial 0.3*f* stress action. The coupling action of an initial stress of 0.3*f* and FTCs in the 6.8% composite salt solution aggravated the damage of FTCs to concrete. When concrete under FTCs in the 6.8% composite solution was subjected to an initial load of 0.3*f*, the loss in the DEM of concrete was 149% larger after 50 FTCs and 115% larger after 100 FTCs than that under FTCs in freshwater without an initial load. From the change rule of the compressive strength and DEM of concrete, it can be concluded that the change in the DEM of the concrete can be used to characterize the change in the compressive strength of the concrete when the concrete is subjected to different initial loads under FTCs in freshwater or in a salt solution.

## 4. Performance of Chloride Ion Erosion in Concrete Samples

**Figure 5:**
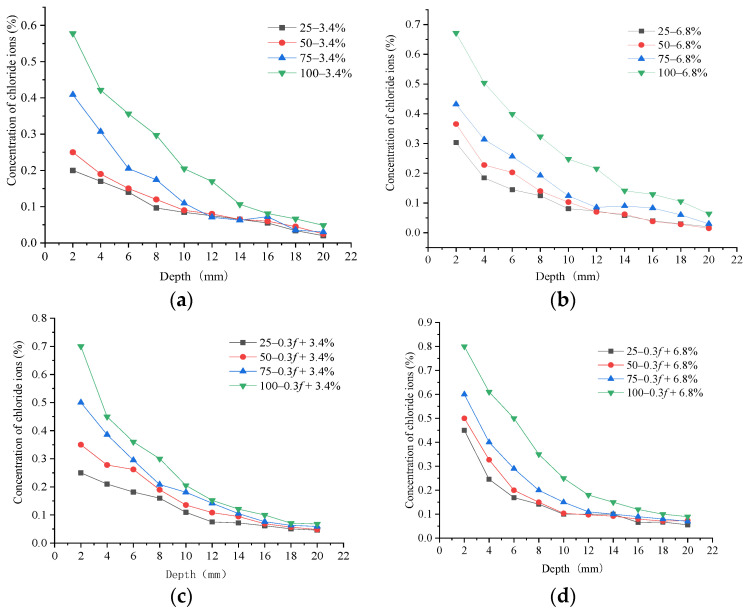
Concentrations of chloride ions at various depths in the concrete samples after various numbers of freeze–thaw cycles. (**a**) After no initial load in a 3.4% composite salt solution. (**b**) After no initial load in a 6.8% composite salt solution. (**c**) After an initial 0.3*f* load in a 3.4% composite salt solution. (**d**) After an initial 0.3*f* load in a 6.8% composite salt solution.

The chloride ion concentrations (CICs) at various erosion depths in the concrete samples under the FTCs in the 3.4% and 6.8% salt solutions were tested for two reasons: (1) to study the erosion characteristics of chloride ions on the concrete materials under salt freeze–thaw environments and (2) to analyze the influence of the initial load on the chloride permeability of the concrete. Before the FTCs in a salt solution, one surface of the concrete samples was designated as a chloride ion erosion surface, and the other five surfaces were coated with epoxy resin. After various numbers of FTCs in the two salt solutions, the erosion surfaces of the concrete samples were ground to different depths. Then, the HC-RCTF chloride ion fast measuring meter was applied to test the CICs at those depths. The mass percentages of chloride ions at the various depths under various numbers of FTCs are shown in Figure 5.

**Figure 6:**
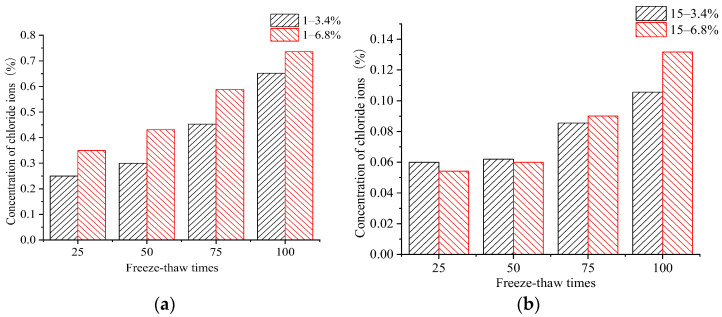
Mass percentage of chloride ions at two depths for various numbers of freeze–thaw cycles. (**a**) 1 mm from the erosion surface. (**b**) 15 mm from the erosion surface.

By comparing the CIC in the samples subjected to the same number of FTCs in the 3.4% and 6.8% composite salt solutions, it can be seen that the concentration of chloride ions in the concrete changed exponentially with the depth. At the same depth, the CIC increased with an increasing number of FTCs. At various numbers of FTCs, the CIC at each depth in the 6.8% composite salt solution exceeded that in the 3.4% salt solution. Samples subjected to an initial load and then FTCs in both salt solution concentrations had a higher CIC than samples without an initial load. This showed that the initial load reduced the impermeability to chloride ions. The change characteristics of the CICs at depths of 1 mm and 15 mm without an initial load under FTCs in both salt solutions are shown in Figure 6.

**Figure 7:**
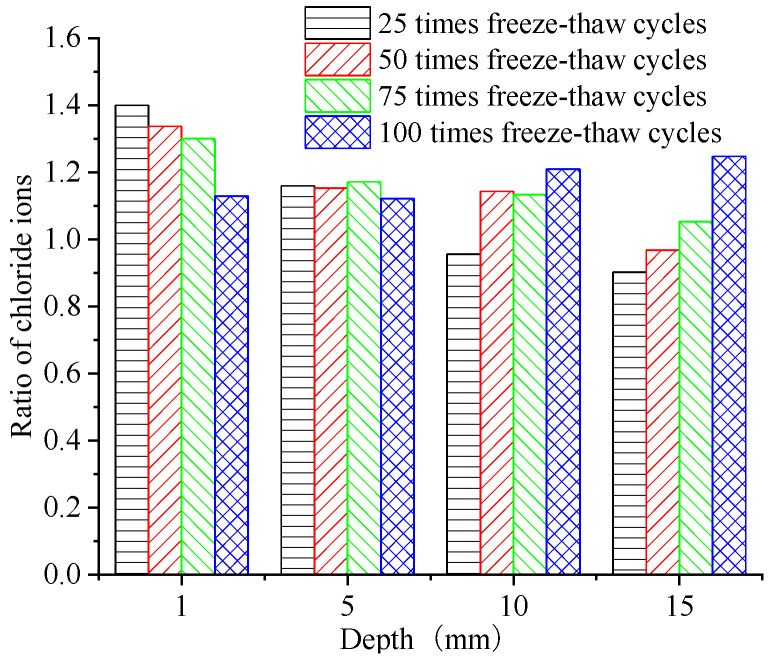
Ratio of the chloride ion concentration at various depths and numbers of freeze–thaw cycles.

It can be seen that the growth rate of the CIC at the erosion surface of the concrete samples was high for various numbers of FTCs in salt solutions, but that growth rate gradually slowed as the depth under the surface increased. The CIC in concrete specimens under FTCs in the 6.8% composite salt solution was larger than concrete specimens under FTCs in the 3.4% composite salt solution. The CIC at a depth of 1mm from the erosion surface was 40% larger after 50 FTCs and 13% larger after 100 FTCs, and the CIC at a depth of 15 mm from the erosion surface was 5% larger after 75 FTCs and 25% larger after 100 FTCs. The rate of the CIC increasing at a depth of 15 mm under 25 and 50 FTCs in both salt solutions was relatively low but under 75 and 100 FTCs, it was obviously high. This demonstrated that the concrete damage started at the erosion surface and gradually developed at greater depths with the increasing number of FTCs. It was found that the chloride impermeability of the concrete samples was gradually reduced with the sustained action of FTCs. The change law of the CIC ratio in concrete samples under various FTCs in the 6.8% and 3.4% salt solutions is shown in Figure 7.

The absolute value of the CIC at the erosion surface increased with the number of FTCs. The difference in the CIC near the erosion surface with the two salt solutions gradually decreased, but deeper under the erosion surface, it gradually increased. When comparing the CIC at the depths of 1 mm, 5 mm, 10 mm, and 15 mm, we found that the ratio of the CIC at depths from 0 to 5 mm after FTCs in the two salt solutions was relatively small, but with the increase in the number of FTCs, the ratio at depths from 10 to 15 mm was larger.

The CIC at a depth of 1 mm from the erosion surface increased by 1.4, 1.16, 0.95, and 0.9 times with the one-fold increase in the salt solution of the FTCs when there were 25, 50, 75, and 100 FTCs, respectively. The CIC at a depth of 10–15 mm from the erosion surface increased with the number of FTCs. The CIC at a depth of 15 mm from the erosion surface increased by 1.12, 1.12, 1.21, and 1.25 times with the one-fold increase in the salt solution of FTCs when the number of FTCs was 25, 50, 75, and 100, respectively. This indicated that in the concrete under FTCs in a high-concentration salt solution, the chloride ions continuously penetrated inside the concrete at the erosion surface, and higher numbers of FTCs increased the penetration rate.

## 5. Pore Structure Characteristics of Concrete Samples

### 5.1. T2 Spectrum Characteristics

**Figure 8:**
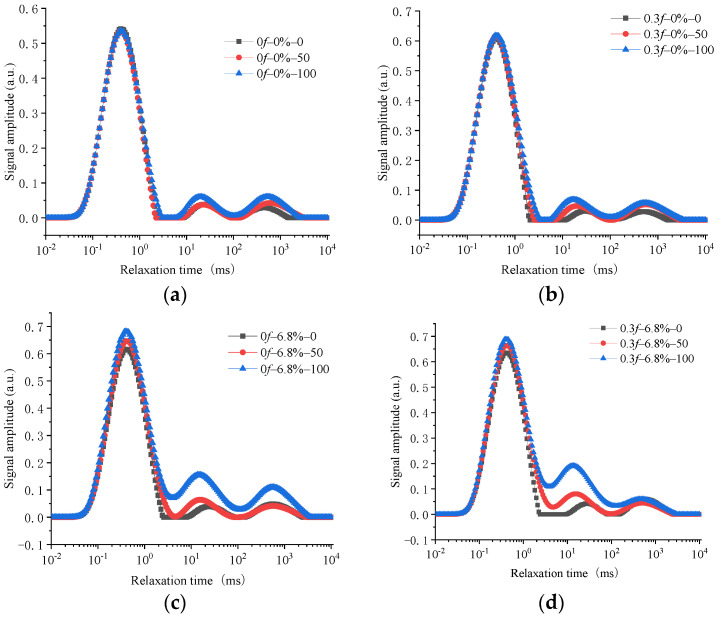
T2 spectra for various relaxation times and numbers of freeze–thaw cycles. (**a**) Without an initial load in the freshwater. (**b**) After an initial load of 0.3*f* in freshwater. (**c**) Without an initial load in a 6.8% composite salt solution. (**d**) After an initial load of 0.3*f* in a 6.8% composite salt solution.

Nuclear magnetic resonance technology has been proven to be an effective, non-destructive testing method for testing pore distribution characteristics and other parameters in cement-based materials [28,29]. In this study, the pore characteristics of the concrete samples were tested by a MesoMR23-060 V-1 nuclear magnetic response (NMR) apparatus, and the Carr–Purcell–Meiboom–Gill (CPMG) sequence was applied to test the NMR T2 spectrum [30]. The transverse relaxation time (T2) of pore water in concrete can be used to measure pore diameter characteristics in concrete structures [31]. The signal intensity of different relaxation times can reflect the pore proportion characteristics inside the material by using inversion [32]. Figure 8 shows the variation properties of the T2 spectrum of concrete samples subjected to no initial load and an initial load of 0.3*f*, and over various numbers of FTCs in freshwater and a 6.8% composite salt solution.

The transverse coordinate in the T2 spectrum represents the relaxation time and is shown by logarithmic coordinates. The relaxation time reflects the diameter of a hole in a material. The longer the relaxation time in the transverse coordinates, the greater the pore diameter. The ordinates in the T2 spectrum represented the signal intensity of the NMR inversion of the pores, and the value of the signal intensity characterized the ratio of the pore diameter in the corresponding transverse coordinate. The larger the ordinate, the larger the proportion of the correspondingly sized pores in the entire material. By comparing the T2 spectra of the concrete samples under various experimental conditions, it could be seen that there were three prominent peaks in the T2 spectra. This indicated that there were three main pore diameters in the structure.

The ordinate values of the T2 spectra of concrete materials increased with an increasing number of FTCs. That is, under FTCs in freshwater and in salt solutions, the total peak area per unit mass gradually increased with an increasing number of FTCs. This shows that the porosity per unit mass of concrete samples increased with the number of FTCs. Comparing the changes in the characteristics of the concrete under FTCs in freshwater and the 6.8% composite salt solution, it was found that the pore volume per unit mass of concrete samples under FTCs in the salt solution was significantly greater than that in freshwater. The T2 spectra area of concrete under FTCs in the 6.8% composite salt solution increased by 31% and 56% more than it did under FTCs in freshwater when the number of FTCs was 50 and 100, respectively. By comparing the influence of the initial load on the value of signal intensity per unit mass of the concrete samples under FTCs in various solutions, it was found that the pore volume per unit mass of concrete samples increased with the increase in initial stress, regardless of whether the FTCs were in freshwater or a salt solution. The T2 spectra area of concrete under FTCs in freshwater subjected to an initial load of 0.3*f* increased by 31% and 56% more than it did under FTCs in freshwater without being subjected to an initial load when the number of FTCs was 50 and 100, respectively.

The second and third peaks increased more obviously in the T2 spectra, which shows that the pores in the pore diameter range corresponding to those peaks obviously changed under FTCs. The initial 0.3*f* stress action caused a larger area of the T2 spectra to undergo a second peak than when there was no initial load. The area of the T2 spectra undergoing a second peak increased by 32% after 50 FTCs and by 20% after 100 FTCs when the concrete was under FTCs in freshwater, and the area of the T2 spectra undergoing a second peak increase by 48% after 50 FTCs and by 32% after 100 FTCs when the concrete was under FTCs in the 6.8% composite salt solution. The FTCs in the 6.8% composite salt solution caused a larger area of T2 spectra to undergo a second peak than FTCs in freshwater. The area of the T2 spectra undergoing a second peak increased by 90% after 50 FTCs and by 200% after 100 FTCs when the concrete did not have an initial load, and the area of the T2 spectra undergoing a second peak increased by 185% after 50 FTCs and by 372% after 100 FTCs when concrete specimens under FTCs in the 6.8% composite salt solution were subjected to an initial 0.3*f* stress action. The coupling action of an initial load of 0.3*f* and FTCs in the 6.8% composite salt solution aggravated the area of the T2 spectra undergoing a second peak. When concrete under FTCs in the 6.8% composite solution was subjected to an initial load of 0.3*f*, the area of the T2 spectra undergoing a second peak was 185% larger after 50 FTCs and 372% larger after 100 FTCs than it was under FTCs in freshwater without an initial load.

The T2 spectra of samples subjected to an initial load of 0.3*f* and which then underwent FTCs were compared to the spectra of unloaded samples. It was found that the second and third peaks of the spectra increased significantly in the loaded samples, as did the relaxation time of the spectra’s transverse coordinates corresponding to each peak. Similar phenomena were found when concrete specimens under FTCs in composite salt solutions were compared to those in freshwater. These showed that the damage of an initial load and FTCs in a composite salt solution increased the effect of FTCs on the damage inside the concrete and made the pores corresponding to the second and third peaks of the T2 spectrum significantly increase.

### 5.2. Ratio of the Peak Area of the T2 Spectrum

The unit mass porosity of the samples was proportional to the number of FTCs, initial load, and salt concentration. This showed that an increase in the number of FTCs, the initial load, and the concentration of the salt solution increased the damage of FTCs on the concrete materials. The damage to concrete samples caused by the FTCs in the 6.8% composite salt solution without an initial load was more severe than it was in freshwater and when subjected to an initial load of 0.3*f*.

**Figure 9:**
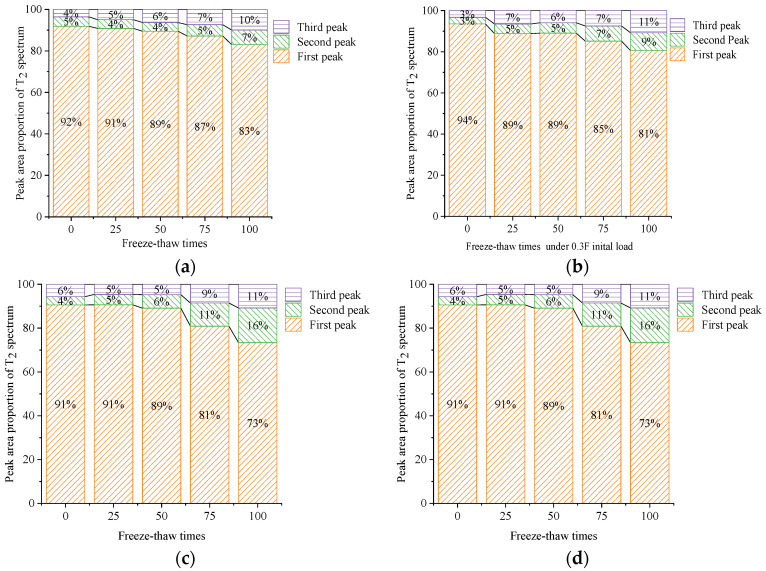
Peak area proportions of T2 spectra. (**a**) Freeze–thaw cycle in freshwater without an initial load. (**b**) Freeze–thaw cycle in freshwater under an initial load of 0.3*f*. (**c**) Freeze–thaw cycle in a 6.8% composite salt solution without an initial load. (**d**) Freeze–thaw cycle in a 6.8% composite salt solution under an initial load of 0.3*f*.

Overall, the FTCs led to the damage inside the concrete; that is, the increase in porosity per unit mass inside the concrete according to the observation from a micro perspective, and the change in the mechanical and physical properties of the concrete samples according to the observation from a macro perspective, showed that the compressive strength and DEM of the concrete samples decreased gradually. Each transverse coordinate of the T2 spectrum corresponded to a special pore diameter, micropores and transition pores (<2.5 ms), mid-sized pores (2.5–100 ms), and large pores and cracks (>100 ms) [33], which can be approximated as the change in three peaks in the T2 spectrum of concrete samples under FTCs. The different changes in the pore ratio of different pore diameters inside the concrete samples under FTCs are shown in Figure 9.

By comparing the change in each peak area of the T2 spectrum of concrete samples under FTCs in different solutions, it can be seen that the proportion of micropores and transition pores in the concrete samples gradually decreased, whereas the proportion of mid-sized pores and large pores and cracks in the samples gradually increased. The rate of the increase in the proportion of mid-sized pores was larger than that of large pores and cracks. The change of pores inside the concrete subjected to FTCs shows that with the increase in the number of FTCs, the compressive strength and DEM of the concrete were closely related to the increase in the proportion of mid-sized pores and large pores and cracks. It was also concluded that from the changes in the peak ratios in the T2 spectrum, the action of the initial load inflicted a certain amount of damage on the concrete samples. The proportion of mid-sized pores inside the unloaded samples under FTCs in the 6.8% composite salt solution increased more than that in freshwater samples subjected to an initial load of 0.3*f*, which shows that the damage caused by the salt solution was more severe than that caused by the 0.3*f* load. This change law of the proportion of pores inside the concrete was consistent with the change characteristics of the compressive strength of the concrete.

## 6. Ingredient Characteristics of Concrete Samples

### 6.1. Principal Component Analysis

**Figure 10:**
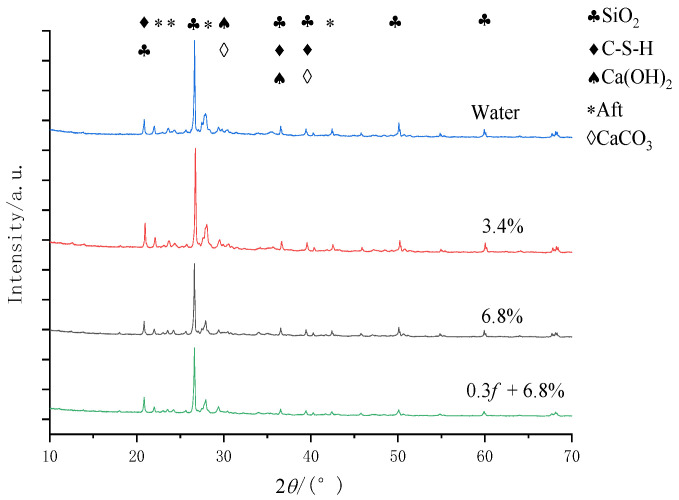
X-ray diffraction spectra analysis of concrete surface erosion after 100 freeze–thaw cycles.

The composition of the concrete materials was observed by an X’Pert^3^ Powder X-ray powder diffractometer. An X-ray diffraction analysis of concrete surface erosion after 100 FTCs is shown in Figure 10. There are several prominent diffraction peaks in the X-ray patterns. They are 20.82° quartz, calcium silicate hydrate (C-S-H) gel, 11.78° gypsum, 18.02° calcium hydroxide, 9.06° and 15.86° ettringite, and 29.36° calcite. After FTCs in the salt solutions, the components of the concrete included silica (SiO_2_), gel (C-S-H,3CaO·2SiO_2_·3H_2_O), calcium hydroxide (CH, Ca (OH)_2_), calcium aluminate hydrate (C3AH6,3CaO·Al_2_O_3_·6H_2_O), and hydrated calcium aluminate sulphate (Aft/AFm). There were NaCl crystals in the composite salt. The calcium carbonate (CaCO_3_) was formed by the reaction of NaHCO_3_ in the composite salt with Ca (OH)_2_ in the cement stone. Hydrate calcium sulphate (CaSO_4_·2H_2_O) was formed by the reaction of sodium sulphate (Na_2_SO_4_) with Ca (OH)_2_ of the cement hydrate. The ettringite (Aft, C3A·3CS·H32,3CaO·Al_2_O_3_·3CaSO_4_·32H_2_O) and hydrated calcium aluminate sulphate (AFm, C3A·CS·H12,3CaO·Al_2_O_3_·CaSO_4_·12H_2_O) were formed by the reaction of hydrated calcium (CaSO_4_·2H_2_O) with tricalcium aluminate (C3A,3 CaO·Al_2_O_3_) in the cement.

### 6.2. Component Characteristics

**Figure 11:**
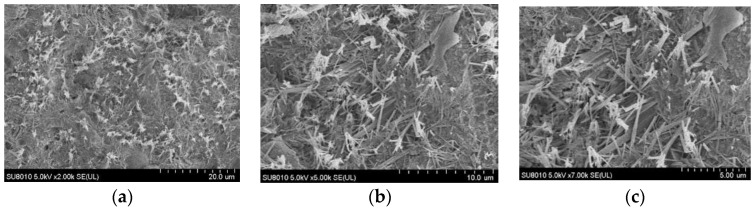
SEM images of concrete before freeze–thaw cycles. (**a**) 2000×. (**b**) 5000×. (**c**) 7000×.

**Figure 12:**
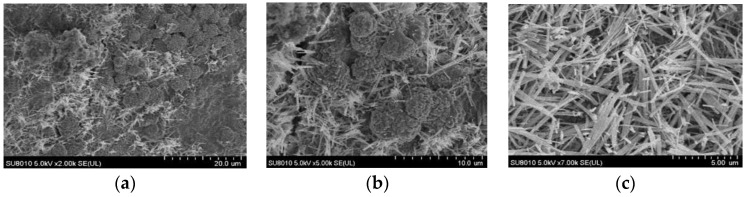
SEM images of concrete after freeze–thaw cycles in a 6.8% composite salt solution with an initial load of 0.3*f*. (**a**) 2000×. (**b**) 5000×. (**c**) 7000×.

An SU8010 positron emission scanning electron microscope (SEM) was used to analyze the microstructure of the concrete samples and the micro-composition characteristics of the samples 2 mm under the erosion surface before and after FTCs in salt solutions. The SEM images of the concrete before the FTCs are shown in Figure 11. SEM images of concrete subjected to an initial load of 0.3*f* and then 100 FTCs in a 6.8% composite salt solution are shown in Figure 12. It can be seen there is a large amount of ettringite (AFt), a needle-like crystal, and flocculent C-S-H gel in the ordinary cement concrete. By comparing the SEM photographs of the two cases, it can be seen that the microstructure of the concrete firmed before being subjected to FTCs. When the concrete samples were subjected to an initial load of 0.3*f* and then to 100 FTCs in a 6.8% composite salt solution, more chloride ions, sulphate ions, and carbonate ions eroded into the concrete and formed crystalline salt. At the same time, the laminate crossed-needle-flake ettringite also increased significantly. At that point, the surface of the concrete samples was no longer flat and became looser, and there were even some penetrating cracks inside the structure. The pores inside the concrete structure increased, which corresponded to the macroscopic performance in which the interface bonding was reduced inside the concrete, which led to the damage andstrength reduction of the concrete under FTCs in a salt solution.

## 7. Correlation Analysis

**Figure 13:**
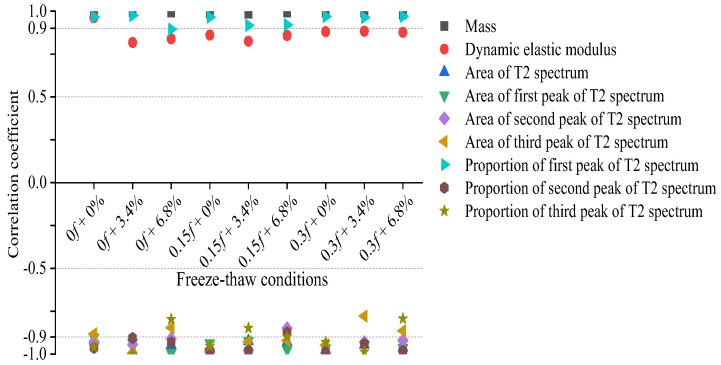
Linear correlation coefficients.

It can be seen from the previous analysis that FTCs led to concrete damage, and changes in the compressive strength, DEM, and the T2 spectrum of the concrete occurred regularly. To study the relationship between the macroscopic mechanical properties of the concrete and the microscopic pore changes inside it, linear correlations were made between the compressive strength of the concrete and the concrete mass, DEM, the area of the T2 spectrum, the areas of the first, second, and third peaks of the T2 spectrum, and the proportion of the first peak. Figure 13 shows the values of the linear correlation coefficients between the compressive strengths of the concrete and the related indicators of the concrete’s properties under the different combined experimental actions.

By comparing the linear correlation coefficients between each index and the compressive strength of concrete, it can be seen that there was a close linear correlation between the change in the compressive strength of the concrete and the concrete mass, DEM, the area of the T2 spectrum, the areas of the first, second, and third peaks of the T2 spectrum, and the proportion of the first peak when the concrete was first subjected to a different initial action with a low load and then to FTCs in different low-concentration salt solutions. The values of most linear correlation coefficients were approximately 0.9, with individual coefficients close to 1. Those results show that the change rules of mass, DEM, and pore characteristics inside the concrete were similar to the change rule of the compressive mechanical properties of concrete samples under the rapid FTCs in different salt solutions. Among the pore characteristics of the T2 spectra of the concrete samples, the change in the proportion of the first peak of the T2 spectrum had a close negative linear correlation with the compressive strength of concrete, and the linear correlation between the two properties of the concrete was relatively stable under the rapid FTCs.

## 8. Conclusions

(1) When the initially loaded concrete samples underwent FTCs in freshwater or salt solutions, internal damage occurred, and more internal cracks were generated. The combined action of the initial load and the salt freeze–thaws led to more severe damage to the concrete.
(2) When concrete samples underwent FTCs in the low-concentration salt solution, the negative effect of the salt produced considerable freezing pressures inside the concrete, which led to more severe damage. The increase in the number of FTCs intensified the concentration of chloride ions, which eroded the concrete samples.
(3) It can be seen from the change in the microscopic structure of the concrete materials under FTCs in freshwater and salt solutions that the overall porosity of the concrete increased with an increasing number of FTCs, and the proportion of mid-sized pores and large pores and cracks gradually increased inside the concrete. During the FTCs, the compressive strength and DEM of the concrete gradually decreased, which had a close correlation with the proportion of the increase in the mid-sized pores and large pores and cracks.
(4) This paper discusses the mechanical, physical, and microscopic pore change properties of ordinary cement specimens under FTCs in a low-concentration salt solution and concrete specimens subjected to low-stress initial loads. There is still a need for further studies on the mechanical, physical, and internal pore morphology changes of high-strength cement concrete or concrete under FTCs subjected to high-stress initial loads.

## Figures and Tables

**Table 1:** Cement concrete mix ratio (kg/m^−3^).

Cement	Water	Fly Ash	Fine Aggregate	Coarse Aggregate	7 d Compressive Strength	28 d Compressive Strength
500	250	55	600	1105	32.8	42.5

**Table 2:** Experimental scheme of freeze–thaw cycles in salt solutions.

Stress Levels	Salt Solution Ratio of Rapid Freeze–Thaw (g/L)			Solution Concentration	Freeze–Thaw Cycles
	Na_2_SO_4_	NaCl	NaHCO_3_		
0, 15%, 30%	0	0	0	0	0, 25, 50, 100
0, 15%, 30%	13.36	7.46	14.38	3.4%	0, 25, 50, 100
0, 15%, 30%	26.72	14.92	28.76	6.8%	0, 25, 50, 100

## Data Availability

Not applicable.
